# Self-regulating photochemical Rayleigh-Bénard convection using a highly-absorbing organic photoswitch

**DOI:** 10.1038/s41467-020-16277-7

**Published:** 2020-05-25

**Authors:** Serena Seshadri, Luke F. Gockowski, Jaejun Lee, Miranda Sroda, Matthew E. Helgeson, Javier Read de Alaniz, Megan T. Valentine

**Affiliations:** 1 Department of Chemistry, University of California Santa Barbara, Santa Barbara, CA 93106 USA; 2 Department of Mechanical Engineering, University of California Santa Barbara, Santa Barbara, CA 93106 USA; 3 Department of Chemical Engineering, University of California Santa Barbara, Santa Barbara, CA 93106 USA

**Keywords:** Photocatalysis, Chemical physics, Chemical engineering

## Abstract

We identify unique features of a highly-absorbing negatively photochromic molecular switch, donor acceptor Stenhouse adduct (DASA), that enable its use for self-regulating light-activated control of fluid flow. Leveraging features of DASA’s chemical properties and solvent-dependent reaction kinetics, we demonstrate its use for photo-controlled Rayleigh-Bénard convection to generate dynamic, self-regulating flows with unparalleled fluid velocities (~mm s^−1^) simply by illuminating the fluid with visible light. The exceptional absorbance of DASAs in solution, uniquely controllable reaction kinetics and resulting spatially-confined photothermal flows demonstrate the ways in which photoswitches present exciting opportunities for their use in optofluidics applications requiring tunable flow behavior.

## Abstract (web summary)

Autonomous control of liquid motion is vital to the development of new actuators and pumps in fluid systems but autonomous control of fluid motion is inaccessible in current systems. Here, the authors identify unique features of a photochromic molecular switch that enables its use for self-regulating light activated control of fluid flow.

## Introduction

Autonomous control of liquid motion is vital to the development of new actuators and pumps in fluid systems. Recently, light has been recognized as a formidable tool for non-invasive, wavelength-selective, remote control over liquid motion with high spatial and temporal resolution. Recent studies have sought to harness light energy for mixing^1,2^, particle arrangement^3,4^, and actuation^5,6^ in solution. Typical systems for such applications make use of photothermal plasmonic nanoparticles and complex nanostructures^7–10^, electrochemically activated substrates^11,12^, or rely on interfacial control to drive fluids^13–16^. Though these current systems provide effective control over flow, autonomous control of fluid motion (i.e., without external manipulation) is inaccessible via the chemistries used in these approaches. Self-regulated fluid pumping is, in its current form, most commonly accomplished via the establishment of local gradients of solutes resulting from a chemical reaction^17,18^. However, many of these systems are limited by the depletion of the chemical source which is used to generate flow. Self-regulation is advantageous where manipulation of flow is desirable without external manipulation or complex chemistry. As such, we investigate here how high-absorbing, negatively photochromic molecular switches might be used to overcome these limitations.

Generally, irradiating highly absorbing particles or molecules in a solution locally heats the solution, and in turn can result in Rayleigh-Bénard convection, in which thermal gradients generate buoyancy-driven flow. However, in the case of such photothermal Rayleigh-Bénard convection under constant, continuous irradiation (i.e., a constant rate of energy input) of an otherwise closed system, establishing a steady state requires a mechanism to inhibit the rate of Rayleigh-Bénard convection as the total energy in the system increases over time. In the case of a high-absorbing, non-bleaching dye or particle, where a photothermal gradient is established, the mechanism of inhibition is the heat loss to the surrounding environment due to heat transfer through the confining walls. Absent this external energy loss, there is no mechanism by which to dynamically decrease the amount of irradiant photon energy that is converted to heat (other than to externally decrease the intensity of incident light). By contrast, the use of negatively photochromic molecules, which can reversibly switch from a high-absorbing and colored state to a non-absorbing and clear state upon irradiation provides an internal photochemical control mechanism: as the light intensity increases, more bleaching occurs, which limits the total amount of irradiant energy that can be converted to heat and enables a tunable temperature gradient. These self-regulatory properties critically rely on the reversibility of the bleaching reaction and its ability to establish and sustain a concentration gradient of the photothermal material and enable spatially varying absorbance in solution that is dynamically maintained. As such, self-regulation can be established and maintained internally by balancing the rates of heat generation and loss through the photoswitching properties of the molecule and heat transfer within the fluid and to the external environment. Furthermore, variable extrinsic factors (e.g., concentration) known to alter characteristics of the photoreaction (e.g., reaction rate) serve as control “knobs” that, in turn, also enable control over the behavior of the Rayleigh-Bénard convection by dynamically tuning the bleaching profile of the photochrome.

**Fig. 1:**
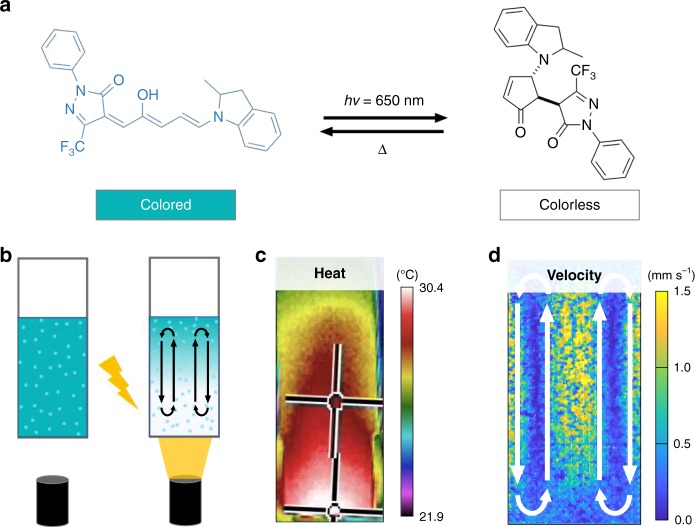
Donor-acceptor Stenhouse adducts for photothermal control of fluid flow. **a** DASA-CF_3_-PI switching under visible light irradiation from an extended, colored, open form to a compact, colorless, closed form. **b** Schematic of experimental setup for convective particle tracking. Light shone on the bottom of a quartz cuvette causes DASAs to switch to the colorless form, while also generating **c** heat gradients and **d** fluid flows due to convection in toluene.

The opportune development of an emerging class of negative photoswitches, donor-acceptor Stenhouse adducts (DASAs), with highly tunable reaction kinetics in a range of solvents allows us to understand and exploit the factors that govern self-regulated fluid flow. DASAs undergo a transformation from a colored, extended open form to a bleached, compact, closed form upon irradiation with visible light (Fig. 1a) and have been leveraged by us^19–22^ and others^23–27^ for their synthetic modularity, wavelength tunability, and negative photochromic properties. In addition, previous studies of DASAs in solution have shown that their switching kinetics are highly dependent on the polarity of the solvent, concentration, and the architecture of the donor and acceptor groups^21,22,25,28^. Herein, we report how combining our understanding of DASAs’ photoswitching kinetics with an ability to control physicochemical conditions such as molarity, solvent, and light intensity enables both controlled and self-regulating fluid motion in organic solutions. The system operates as follows: by irradiating a solution containing DASA with visible light (Fig. 1b) a controlled bleaching front (i.e., a growing non-absorbing region) is generated, which enables the local manipulation of solution temperature (Fig. 1c) which in turn incites a time-varying thermal gradient and thus Rayleigh-Bénard convection (Fig. 1d). These convective flows are inherently self-regulated via the complex interplay between heat transfer within the fluid and to the external environment, the photochemical conversion kinetics, and the high absorption—enabling a novel method of control over fluid motion. The careful manipulation of the reaction kinetics of a photoswitch uniquely enables the bleaching front progression to dictate the flow behavior observed in solution. This degree of control is presently inaccessible via other methods and, thus, using a negatively photochromic molecular photoswitch such as DASA opens new avenues for the use of high-absorbing photoswitches in applications for self-regulated fluid pumping and mixing.

## Results

### DASA switching kinetics

**Fig. 2:**
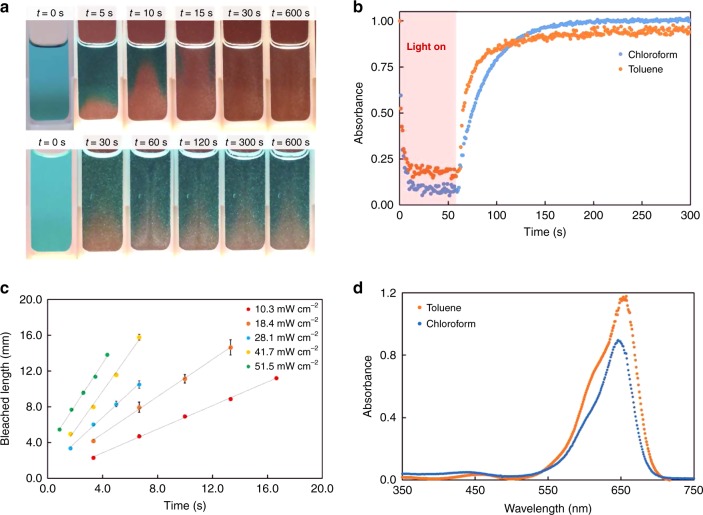
Photochemical properties of DASA-CF_3_-PI in chloroform and toluene. **a** Bleaching front progression through 0.25 mM DASA-CF_3_-PI in chloroform stabilized with 0.75% ethanol (top) and toluene (bottom) irradiated with 25 mW cm^−2^ light. **b** Reversible switching kinetics of DASA-CF_3_-PI in chloroform and toluene at 10 μM. **c** Bleaching front length versus time for 0.125 mM DASA in chloroform, where the slope provides a measure of the front velocity. **d** Absorbance of DASA-CF_3_-PI in toluene and chloroform at 14 μM.

In order to drive dynamic, photochemically regulated fluid flows we focused on the newly developed third generation DASA bearing a CF_3_ pyrazolone-based acceptor and a 2-methyl indoline donor^22^ (referred to hereafter simply as DASA-CF_3_-PI) due to its high molar absorptivity and tunable forward and reverse switching kinetics in organic solvents. Though DASA-CF_3_-PI switches from its colored, open form to a bleached, closed form (Figs. 1a, 2a) in both toluene and chloroform, the tunability of this behavior arises from solvent- and concentration-dependent photoswitching kinetics. The following studies highlight the dynamic control of flow behavior by varying solvent, concentration, and light intensity. Specifically, this DASA derivative exhibits a faster backward photoreaction in toluene than in chloroform stabilized with 0.75% ethanol, as evidenced by reverse rates of reaction (*k*_back_) of 0.046 s^−1^ and 0.035 s^−1^ in toluene and chloroform, respectively, at 10 μM (Fig. 2b, Supplementary Note 1). This difference in *k*_back_ is intensified at high concentrations—as DASA-CF_3_-PI exhibits a stronger concentration-dependence in toluene than in chloroform—leading to an even faster back reaction^29^. The bleaching front velocity (Fig. 2c) and solvent-dependent switching combined with DASA’s high molar absorptivity (Fig. 2d), can be exploited to generate sharp thermal gradients at the interface between the bleached and non-bleached portions of the solution that drive convective flows (Fig. 2a). Varying light intensity also has an effect on the magnitude of observed thermal gradients.

### Molar absorptivity drives thermal gradient

**Fig. 3:**
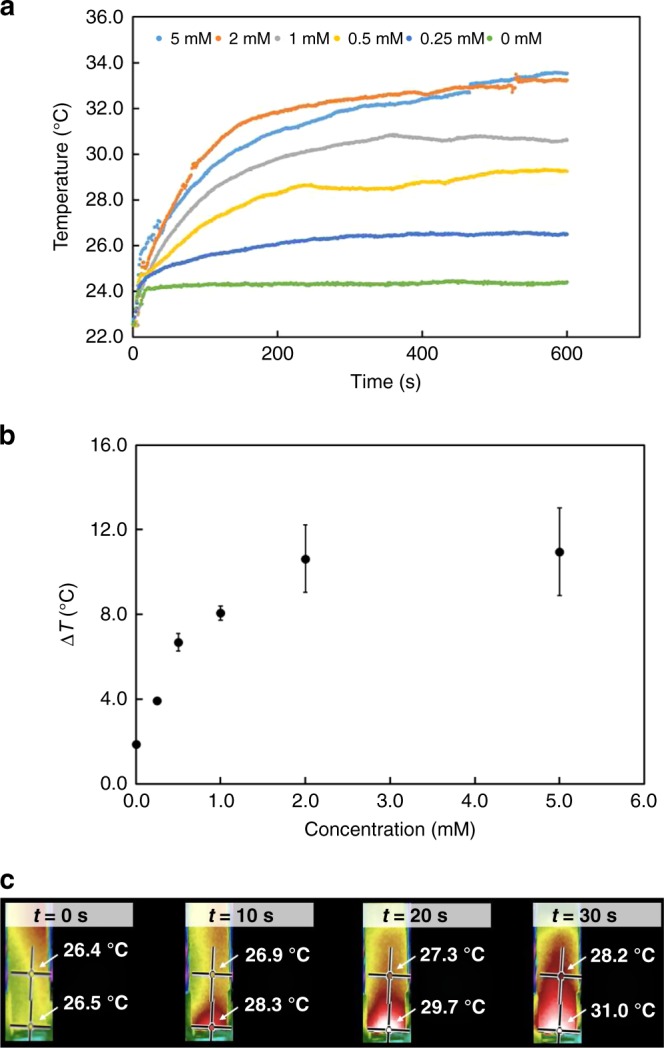
Photothermal temperature changes due to DASA-CF_3_-PI in solution. **a** Average heating of chloroform solutions with varying concentrations of DASA-CF_3_-PI using 214 mW cm^−2^ white light. **b** Change in temperature as a function of DASA-CF_3_-PI concentration from *t* = 0 s to *t* = 600 s. Average values are plotted with error bars calculated from the standard deviation of replicate measurements (*N* = 3). **c** Thermal images of heat gradient established in the first 30 s of irradiation of 2 mM DASA-CF_3_-PI in chloroform, as measured with an IR camera. Note, the camera’s auto-adjust feature prevents the use of a consistent legend for the temperature, so numeric values for two locations are shown.

In its colored form, the high molar absorptivity of DASA enables the effective conversion of light energy to heat, which in turn drives fluid motion. Using UV/Vis spectroscopy, we determined the molar absorptivity of DASA-CF_3_-PI in toluene, chloroform, and dichloromethane to be 118,820 ± 516 M^−1^ cm^−1^, 88,867 ± 300 M^−1^ cm^−1^, and 129,190 ± 490 M^−1^ cm^−1^, respectively (Supplementary Fig. 2). For comparison, we determined the molar absorptivity of highly absorbing, commercially available organic dye Nile Red to be 38,000 M^−1^ cm^−1^ and that of a non-bleaching DASA analog to be 70,500 M^−1^ cm^−1^ (Supplementary Fig. 3). To demonstrate the dramatic changes in temperature enabled by the absorbance of DASA-CF_3_-PI, solutions of varying concentration in chloroform were irradiated for ten minutes using a 214 mW cm^−2^ white light source (Fig. 3a). From these experiments, we observe 2 °C temperature changes in solvent without dye due to the heat from the light source and up to 12 °C in the presence of DASA-CF_3_-PI (Fig. 3b). Furthermore, infrared imaging illustrates a temperature gradient established throughout the solution within tens of seconds of irradiation (Fig. 3c). Above 2 mM, however, increasing the DASA-CF_3_-PI concentration fails to further increase the solution temperature, as a rapid back reaction impedes the penetration of light and thus a similar bleaching front is established at these concentrations.

### Control of bleaching front

Using the experimental configuration depicted in Supplementary Fig. 1, we found that solutions of DASA-CF_3_-PI in chloroform at low concentrations (e.g., 0.25 mM) exhibit a well-defined bleaching front (i.e., a growing zone of bleached solution) that propagates through the entire volume of the solution similar to those previously studied^30^. However, this behavior is different from that in toluene, where the bleaching front begins to propagate through the solution but is then halted at some non-zero height due to a competitive back reaction (Fig. 2a). Understanding the bleaching behavior is critical, as DASA’s absorbance—and thus, its ability to generate heat—falls steeply once it has transitioned from the colored to colorless state. The time- and length-scales over which these solutions bleach can be analyzed to predict characteristics of the flow behavior and tune the system for self-regulation.

The bleaching profile corresponds to a sharp gradient in concentration of the open form of DASA-CF_3_-PI between a bleached zone (A) and unbleached zone (B). Because of the large disparity in photoabsorption efficiency between the open and closed forms of DASA-CF_3_-PI (Fig. 2b), the heat generation that drives the convective flow is primarily produced in the unbleached (i.e., colored) zone where the concentration of the open form is significantly greater than the closed form. As such, we expect that the total rate of energy input that drives photothermal convection will be proportional to the average net rate of photoreaction in the bleached zone and the fraction *f* of the container volume occupied by the unbleached zone, $f = \left( {H - h_I} \right)/H$, where *H* is the total fluid height and *h*_*I*_ the height of the bleaching front (i.e., colorless zone). Therefore, to a first approximation, we also expect that the time variation of the magnitude of the maximum convective velocity will qualitatively track with the speed at which the bleaching front advances—i.e., $\it {\mathrm{d}}v_{\mathrm{max}}/\mathrm{d}t \propto \mathrm{d}f/\mathrm{d}t \propto - \mathrm{d}h_I/\mathrm{d}t$. It is therefore of critical importance to understand the kinematics of the bleaching front, and the influence of the various coupled transport processes present in the system.

The velocity of the front depends on the net rate of reaction (forward and backward), mass transport to and from the bleaching front, and convection due to flow and diffusion. By restricting the analysis to only the bleaching front itself, we expect that concentration gradients in the bleached and unbleached regions will be small, such that the diffusive flux is negligible compared with the convective flux. Making this and other simplifying assumptions and solving for the interface velocity yields Eq. (1) (full derivation, Supplementary Note 2).

1 $${\boldsymbol{v}}_I = {\boldsymbol{v}}_{\mathrm{fluid}} + \frac{{\delta r_{I,{\mathrm{open}}}}}{{\Delta N_{I,\mathrm{open}}}}$$

Here $\Delta N_{I,\mathrm{open}} = N_{{\rm{A,open}}} - N_{{\rm{B,open}}}$ is the change in open DASA-CF_3_-PI molar concentration across the bleaching front, $\delta$ is the thickness of the transition in concentration between the bleached and unbleached regions, and $r_{I,\mathrm{open}}$ is the net rate of the reaction, defined in Eq. (2)^29^.

2 $$r_{I,\mathrm{open}} = \sigma ( {N_{I,\mathrm{open}}} ) \cdot \phi _{\mathrm{OC}}( {N_{I,\mathrm{open}}} ) \cdot I( {h_I} ) \cdot N_{I,\mathrm{open}} - k_{\mathrm{back}}( {N_{I,\mathrm{open}}} ) \cdot [ {N_{\mathrm{o}} - N_{I,\mathrm{open}}} ]$$

Note that the forward reaction rate is governed by the absorption cross-section $\sigma$, the quantum efficiency for photoconversion between the open and closed form $\phi _{\mathrm{OC},}$ the irradiation intensity at the bleaching front *I*, and the concentration of open DASA-CF_3_-PI at the bleaching front $N_{I,\mathrm{open}}$. The back reaction is dictated by the rate constant of the backward photoconversion $k_{\mathrm{back}}$ and the concentration of the closed form of DASA-CF_3_-PI ($N_{\mathrm{o}} - N_{I,\mathrm{open}}$). The reverse rate constant is calculated by fitting the experimental absorbance recovery of DASA after irradiation is ceased to a decaying exponential function^29^. Substituting Eq. (2) into (1) results in Eq. (3).

3 $${\boldsymbol{v}}_I = {\boldsymbol{v}}_{\mathrm{fluid}} + \frac{\delta }{{\Delta N_{I,\mathrm{open}}}} \{ {\sigma \cdot \phi _{\mathrm{OC}} \cdot I\left( {h_I} \right) \cdot N_{I,\mathrm{open}} - k_{\mathrm{back}} \cdot [ {N_{\mathrm{o}} - N_{I,\mathrm{open}}} ]} \}$$

Eq. (3) reveals the unique features that are imparted to the photothermal convection due to a reversible photochemical reaction such as that enabled by DASA-CF_3_-PI. When $k_{\mathrm{back}}$ is small (e.g., in chloroform) the velocity of the bleaching front (which is distinct from, but is influenced by, the underlying convective fluid velocity, as indicated in Eq. (1)) is always positive and the bleaching front will grow to fill the volume of the container. Once completely bleached, no further energy due to photothermal processes is generated—allowing the thermal gradient to dissipate and convective flow to be “turned off.” By contrast, if $k_{\mathrm{back}}$ is sufficiently large (e.g., in toluene) the location of the bleaching front can reach a dynamic steady state dictated by the self-regulating balance between (1) the photothermal convection and forward reaction in the unbleached zone, and (2) the back reaction in the bleached zone. Thus, using toluene and chloroform, we demonstrate distinct flow behaviors that take advantage of differences in DASA’s back reaction rates. Unlike other photothermal systems, DASA’s solvent-dependent back reaction kinetics offer unique modular control over dynamic and self-regulating flows in solution.

### Nonlinear relationship between concentration and fluid velocity

Examining the fluid motion in quartz cuvettes containing identical concentrations of DASA-CF_3_-PI to those used in the temperature studies, we observed a nonlinear relationship between the strength of convection at early times and DASA-CF_3_-PI concentration. This relationship arises as a result of the coupled relationship between the back-reaction kinetics of DASA-CF_3_-PI and Beer’s Law. As solutions containing DASA-CF_3_-PI are irradiated with visible light, the drastic changes in temperature depicted in Fig. 3a incite buoyancy-driven Rayleigh-Bénard convection. The strength of this convection can be analyzed by quantifying the speed of flows generated in solution using particle image velocimetry (PIV) to track the motions of neutrally buoyant silica spheres (average diameter = 10 μm). Specifically, 400 μL of solution was pipetted into a cuvette, allowed to settle in the absence of light, and then illuminated from below and recorded using a digital camera (Supplementary Movie 2). Velocity fields were then calculated from the video using open source PIV software (Supplementary Figs. 4 and 5)^31^. These experiments allow us to quantify DASA’s remarkable ability to increase fluid motion in a given solution to several mm s^−1^ using only millimolar concentrations of DASA-CF_3_-PI and commercially available solvents.

**Fig. 4:**
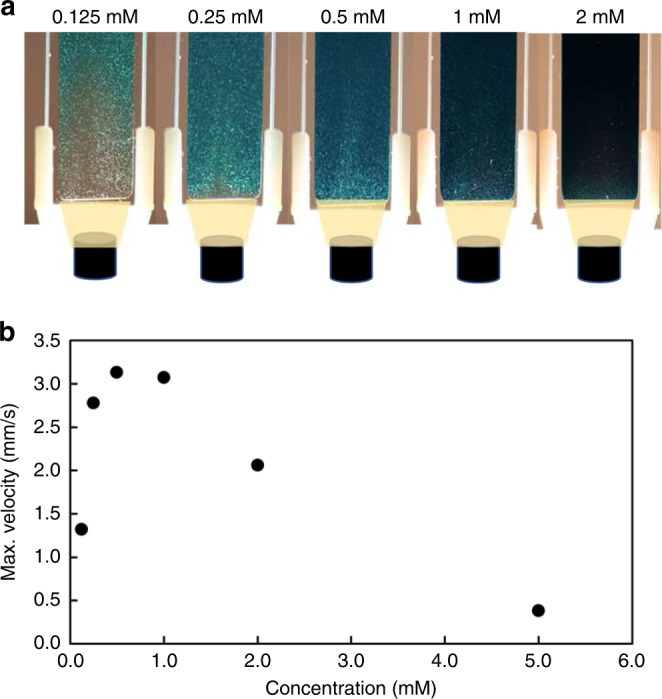
Non-linear concentration dependence of particle velocity. **a** Concentrations ranging from 0.125 to 2 mM of DASA in toluene shown from left to right after 10 s of irradiation with 46 mW cm^−2^ light. **b** Particle velocities as a function of concentration show the nonlinear dependency on concentration. Above 0.5 mM, the dark solution exhibits a masking effect that limits light penetration and, as a result, limits particle velocity.

Although DASA demonstrates efficient photoswitching capabilities in both toluene and chloroform at low concentrations, the rate of back reaction drastically increases above an optimal concentration^29^. This increased rate implies the rapid reversion of DASA-CF_3_-PI molecules from a closed, colorless to an open, colored form even in the presence of constant light. With most of the species in the high-absorbing open form, a masking effect is observed (Fig. 4a) which limits light penetration in the depth of the solution and thus the population of irradiated/switched species. This effect is reflected in the PIV analysis as a continuous decrease in maximum fluid velocity with increasing concentration of DASA-CF_3_-PI (Fig. 4b). As a result, there exists an optimal concentration for which light can fully penetrate a solution to establish appreciable convective flow. We estimate this concentration to be 0.5 mM, where the convective flow reaches maximum fluid velocity of ~3.1 mm s^−1^. While differences in experimental conditions make direct comparison challenging, this fluid velocity is orders of magnitude higher than those reported for common plasmonic particle systems which sustain micrometer per second velocities at similar photon intensity^7,14^. These experiments establish that light attenuates exponentially as the concentration of the absorbing species increases, and highlight concentration as yet another tunable parameter by which fluid velocity may be controlled.

### Bleaching front dictates self-regulating fluid motion

The nonlinear coupling of both solvent- and concentration-dependent photoswitching kinetics and high absorption properties enables unprecedented tunabilty and self-regulation of photothermal flows. Irradiating a solution of a given concentration and solvent incites a photothermally driven flow which creates a dynamic equilibrium at the bleaching front in which the consumption of photoreactants is stoichiometrically replenished by the flux of regenerated photoreactants from the bleached zone. To illustrate this balance, we present two distinct self-regulating flow behaviors using 0.25 mM solutions of DASA-CF_3_-PI in chloroform and toluene, respectively, irradiated using variable light intensity (Supplementary Figs. 6 and 7).

**Fig. 5:**
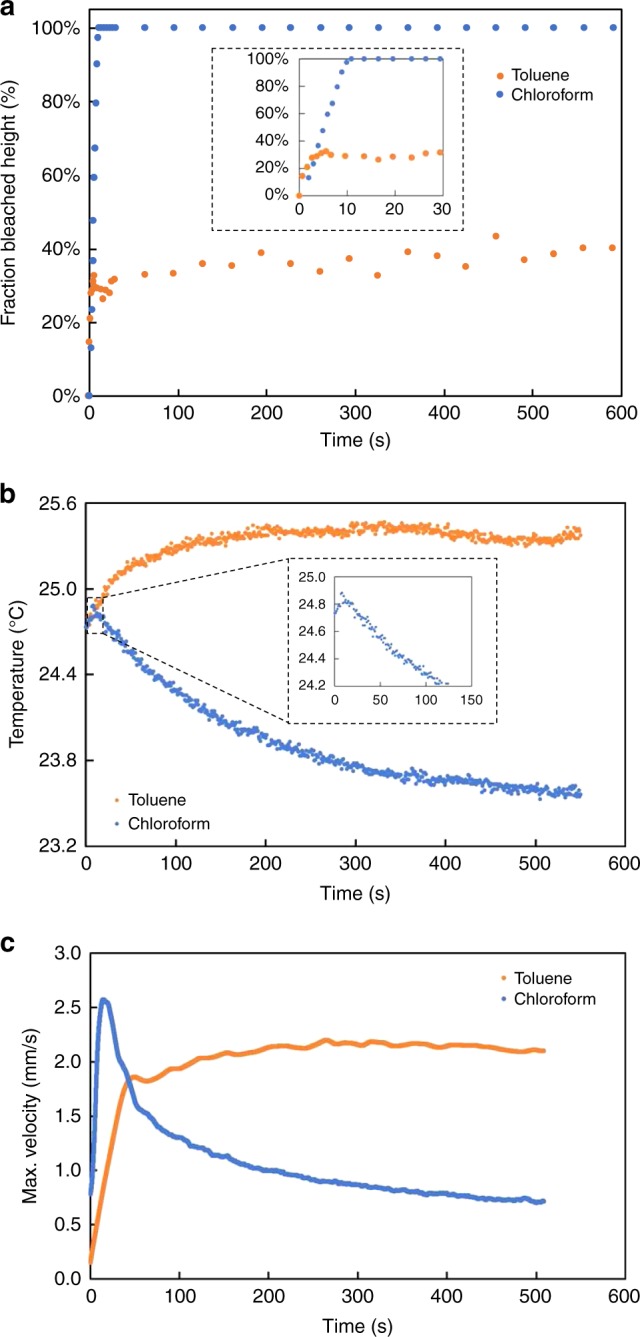
Comparison of fluidic and thermal behavior of 0.25 mM DASA in chloroform and toluene irradiated with 46 mW cm^−2^ white light. **a** Percent of bleached volume (i.e., height of the bleach front / total wetted length) versus time. Inset depicts the same curves from 0 to 30 s. **b** Temperature changes over time for toluene and chloroform. Inset depicts the initial spike and then decline in temperature exhibited by DASA in chloroform. **c** Maximum velocity vs. time plots illustrate characteristic velocity trends. See Supplementary Note 3.

In chloroform, an effective self-extinguishing behavior enabled by the careful tuning of the reaction kinetics of DASA-CF3-PI dictates the self-regulating flow behavior. Specifically, the rate of forward reaction is significantly greater than that of the reverse reaction—allowing the bleaching/photoswitching front to propagate through the solution unimpeded. As such, the bleached zone will eventually grow to fill the fluid volume, and the photothermal convection will cease after the thermal gradient dissipates—providing an effective “off-switch” for the photothermal convective flows, even as the light remains on (Figs. 2a, 5c). The time at which the “switch” occurs can be approximated from the bleach front velocity using Eq. (3) and changes in temperature. In the first eight seconds, the temperature of the solution rises as the bleaching front progresses through the cuvette (Fig. 5b, inset). At roughly the same time (~10 s), the maximum velocity and the maximum temperature difference are achieved. The brief, initial rise in temperature (Fig. 5b, inset) is due to the high absorbance of DASA that initially exist in the open form. However, as the solution bleaches (Fig. 5a) and the light input remains constant, the temperature gradually decreases and eventually plateaus. This characteristic behavior in chloroform—i.e., a steep rise followed by a decrease in maximum velocity and temperature—occurs irrespective of light intensity (Supplementary Fig. 7), although the intensity variations enable control of fluid flow dynamics. Increased light intensity leads to faster absorption and thus higher peaks in maximum velocity, but also a faster bleaching of the DASA solution. This repeatable behavior combined with control over tunable parameters (e.g., light intensity), make this type of switch uniquely amenable to systems in need of dynamic pumping under constant light irradiation and/or spatially precise zones of light penetration. The self-extinguishing behavior is achieved without additional stimuli or changes in concentration and can thus be leveraged for applications that require a fluidic feedback loop.

By contrast, in toluene, where $k_{\mathrm{back}}$ is sufficiently fast and $N_{I,\mathrm{open}}$ is large everywhere, the front accelerates due to both the large net forward reaction and induced photothermal convection due to absorption by the open form. The rate of the forward reaction (dictated by the quantum yield of DASA-CF_3_-PI in toluene) is less than the rate of back reaction, however, the convective fluid velocity term ($v_{\mathrm{fluid}}$) is balanced by the reaction velocity term. This balance results in a static bleaching front (i.e., *v*_*I*_ = 0, see Fig. 5a), which in turn allows for a steady state thermal gradient, and thence a constant fluid velocity that is maintained over ten minutes (Figs. 2a, 5c). In measuring the temperature changes in the system, we similarly find an initial increase in temperature of approximately 0.6 °C within the first two minutes that rapidly stabilizes to a steady-state value for over ten minutes of study. This self-regulation is facilitated by the reversibility of the photoreaction of the DASA-CF_3_-PI system and a matching of forward and backward reaction kinetics. Steady-state flows can also be achieved with other high-absorbing dyes that are not photochromic (Nile Red and non-switching DASA), as well as less optimal photochromic DASA derivatives (Supplementary Figs. 8 and 9). However, the rate and magnitude of convective flow is enhanced, and spatial control enabled, when a highly absorbing negative photochromic molecule is employed. Moreover, the tunable fluid behavior enabled by the DASA-CF_3_-PI system—i.e., a dynamically stabilized fluid velocity—is amenable to fluid systems in need of controlled, constant velocity. Further, the magnitude of the velocity plateau can be adjusted by tuning the light intensity (Supplementary Fig. 7).

### Localized fluid motion

**Fig. 6:**
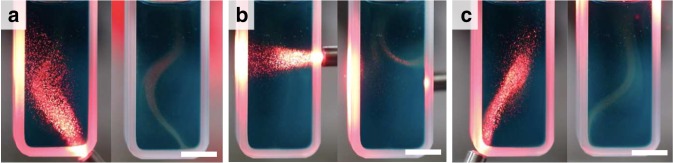
Spatially precise convective flows. **a**–**c** (left panels) Irradiating one portion of the cuvette using a red LED light. **a**–**c** (right panels), convective flows that ensue due to irradiation. The same solution was used for all experiments depicted above (Supplementary Movie 3).

By localizing the light intensity to a point of interest, we create a pathway by which particles can be advected via a spatially addressed photothermal gradient. To demonstrate this effect, we show the locally directed motion of particles using a red LED (*λ* = 617 nm, 21 mW cm^−2^) to illuminate a 0.25 mM solution of DASA-CF_3_-PI in chloroform. Visual inspection of particle trajectories demonstrates that we can direct particles along the path of light propagation with just a few seconds of irradiation (Fig. 6, Supplemental Movie 3). Due to a heat-induced decrease in local fluid density, buoyancy effects enable spatially localized flow, irrespective of the spatial orientation of the light source. As a result of these buoyancy effects, we see from the bleaching that an upward flow is established within seconds after the light source has been removed. The fast reverse kinetics allows for the color to return almost immediately, thus enabling the use of the same solution to direct particles along multiple trajectories.

## Discussion

By leveraging our understanding of DASA’s reversible switching kinetics, we have demonstrated the ability to harness the photochemical response of a negatively photochromic switch to control bleaching and consequently photo-thermal gradients in differing solvents to drive programmed fluid motion. The tunable rate of the back reaction of DASA offers a unique handle by which we can control the self-regulatory properties of the system. Additionally, we can estimate the timescale over which a maximum velocity may be achieved as a function of the relative rates of the photochemical reaction and the propagation of the thermal gradient it produces. As such, we unlock a powerful mechanism by which fluid motion can be controlled using a negatively photochromic molecular switch with highly tunable reaction kinetics. For solvents in which DASA-CF_3_-PI has a fast back reaction, the dynamic steady state achieved in solution produces a self-regulating thermal gradient that can drive fluid flow at a constant velocity. In solvents in which DASA bleaches rapidly and has a slow back reaction, it acts as an effective “off-switch” after the solution has entirely bleached and the thermal gradient dissipates. To our knowledge, this is the first system to demonstrate a self-regulatory degree of control as a result of a photochemical reaction. With careful tuning of reaction kinetics, other high-absorbing negatively photochromic switches can be similarly tailored, providing access to self-regulating control using different wavelengths of irradiation and solvents (Supplementary Fig. 9). This versatile control and tunability provide unrivaled access to fluidic applications requiring high fluid velocities (~mm s^−1^), controllable fluid behaviors, and operation in a range of chemical environments—all achievable using low intensity visible light.

## Methods

### Chemical selection

Chloroform stabilized with 0.75% ethanol and toluene (99.8%) were purchased and used without further purification from Sigma Aldrich. Donor-acceptor Stenhouse adducts were synthesized as described in the procedure previously reported in literature^22^. For each experiment, the compound was dissolved in solutions from fresh bottles of chloroform stabilized with ethanol or toluene and diluted to the desired concentrations.

### Light source

A Schott Ace 150 W fiber optic white light source was chosen for all experiments herein due to its broad coverage of the absorbance spectrum of DASA-CF_3_-PI and other molecules discussed in this work (Supplementary Fig. 3). In addition, it supplied light fluxes otherwise inaccessible with the use of LEDs available to us.

### Characterization

Molar absorptivity of photoswitches and dyes were calculated using spectra obtained with UV/Vis spectrophotometry in triplicate for five concentrations of dye/switch in solvent. Each stock concentration of DASA was prepared 24 h in advance in order to ensure that the photostationary state was reached. All measurements were taken using a standard quartz cuvette with 1 cm pathlength. The photoinduced optical absorption kinetics were measured using a pump-probe setup following previously reported procedures^22^.

### Temperature analysis

Heat changes were obtained using a Phidget 4-Input Temperature Sensor in conjunction with the K-Type Teflon Bead Probe Thermocouple (accurate to ±2 °C). Temperatures were measured at the top, middle and bottom of the cuvette to calculate the absolute and temperature differentials over the course irradiation. Infrared images were obtained using a FLIR E60 IR camera accurate to ±2% of reading.

### Particle tracking and analysis

For all particle tracking experiments, neutrally buoyant hollow spherical silica particles (*D*_avg_ = 10 μm, TSI Inc.) were added to 400 μL of solution in a quartz cuvette (pathlength 2 mm, inner dimensions: 40 × 12 × 2 mm^3^). Samples were irradiated 2 cm from the bottom at which distance the maximum irradiance was determined to be 214 mW cm^−2^. All samples were irradiated with a Schott ACE Halogen Light Source with intensities determined using a Field Max II TOP Laser Power Energy Meter. Videos were then divided into grayscale images using MATLAB code and analyzed using open-source particle image velocimetry software (see Supplementary Methods).

## Supplementary information

Supplementary Information

Description of Additional Supplementary Files

Supplementary Movie 1

Supplementary Movie 2

Supplementary Movie 3

## Data Availability

The authors declare that all relevant data supporting the findings of this work are available from the corresponding authors on request.
