# Supplementary material for: Self-regulating photochemical Rayleigh-Bénard convection using a highly-absorbing organic photoswitch
Source: Nat Commun. 2020 May 25;11:2599. doi: 10.1038/s41467-020-16277-7 (PMC7248117; doi:10.1038/s41467-020-16277-7)
Supplement: Supplementary file 1 — Supplementary Information [file 41467_2020_16277_MOESM1_ESM.pdf]

Supplementary Materials for

Self-regulating photochemical Rayleigh-Bénard convection using a highly-absorbing organic photoswitch

Serena Seshadri[†1], Luke F. Gockowski[†2], Jaejun Lee[1,2], Miranda Sroda[1], Matthew E. Helgeson[3], Javier Read de Alaniz[*1], Megan T. Valentine[*2]

[1]Department of Chemistry, University of California Santa Barbara, Santa Barbara, CA 93106
*Correspondence author email: javier@chem.ucsb.edu

[2]Department of Mechanical Engineering, University of California Santa Barbara, Santa Barbara, CA 93106
*Correspondence author email: valentine@engineering.ucsb.edu

Contents

Supplementary Methods 3
Imaging 3
Particle Image Velocimetry 3
Supplementary Note 1 4
Supplementary Note 2 4
Supplementary Note 3 5
Supplementary Figure 1 7
Supplementary Figure 2 8
Supplementary Figure 3 9
Supplementary Figure 4 10
Supplementary Figure 5 11
Supplementary Figure 6 12
Supplementary Figure 7 13
Supplementary Figure 8 14
Supplementary Figure 9 15
Supplementary References 16

Supplementary Methods

Imaging

A Canon Rebel SL2 (100 mm f/2.8 Macro USM fixed lens, 1x magnification, 29.97 frames per second) was used to image photoreactions occurring in the UV/Vis quartz cuvette. The setup for these reactions is depicted in Supplementary Figure 1. Note that we define the positive *u*-direction to be against gravity (Supplementary Fig. 1). This is later relevant in Supplementary Note 2.

Particle Image Velocimetry

Video records of photoreactions inside the UV/vis cuvette were then segmented into individual grayscale frames based on the camera's frame rate (i.e., 29.97 FPS) using MATLAB code. These frames were then used to perform particle image velocimetry (PIV) using open-source code from Thielecke and Stamhuis.[1,2] Prior to analysis, the region of interest was specified as a rectangle inside of the wetted region of the cuvette where flows occur (Supplementary Fig. 4). Next, a fast-Fourier transform (FFT) window deformation algorithm was selected and four interrogation areas of decreasing window size (i.e., 64, 32, 16, and 8 pixels) were used to analyze the motion of particles (Supplementary Fig. 4), according to published protocols.[3]

The published protocols also recommend post-processing of data to remove erroneous vectors and ensure accurate calculation. This is achieved by surveying a scatterplot of the horizontal and vertical velocities, and removing calculated vectors determined to be outliers. Graphically, this is achieved by selecting a boundary outside of which the data are outliers and will be removed (Supplementary Fig. 5). Visually, the incongruity of these erroneous vectors is confirmed by surveying plots of vector fields overlaid on each analyzed image.

The remaining velocity data were interpolated to provide values at all coordinate points. The data for each frame were then exported as text files containing four columns: the *u*-coordinate, *v*-coordinate, and associated *u*- and *v*-velocities of the fluid. These data were then analyzed using separate, custom-written MATLAB code that analyzes the positive *v*-velocities (i.e., opposing gravity) of each frame, and stores the highest value—i.e., the maximum velocity—at each point in time. These values of maximum velocity are then plotted against time to produce the curves in Supplementary Figs. 6 and 7.

Supplementary Note 1

Studies of the backward photoreaction of DASA 3.0 in toluene and chloroform are restricted to dilute solutions of DASA 3.0 due to the high extinction coefficient of DASA and the limitations of the apparatus detector. Note that here and through the Supplemental Materials, DASA 3.0 refers to third generation DASA bearing a CF_3 pyrazolone-based acceptor and a 2-methyl indoline donor[6] (also referred to as DASA-CF_3-PI).

Supplementary Note 2

In general, the shape and location of the concentration gradient in the bleaching front depends in a complex way on the illumination profile in the container, the resulting bleaching reaction kinetics, and the convective mass transport of DASA 3.0, which is carried along by the flow produced as a result of the Rayleigh-Benárd convection. However, if all we seek is the location of the front and its motion in time, then the problem can be simplified by restricting the analysis solely to the bleaching front itself.

Consider that the bleached zone near the bleaching front is maintained at a relatively constant concentration $N_{A,open}$ of open DASA 3.0, and that the unbleached zone near the bleaching front is maintained at a relatively constant concentration $N_{B,open}$. For simplicity, we assume that the concentration varies from $N_{A,open}$ to $N_{B,open}$ over a distance, δ, that is much smaller than both the height of the bleaching front h_I and the fluid height *H*. Under this assumption, the bleaching front can be treated as a fictitious interface between "phases" A and B, at which a step change in N_{open} occurs due to the photoreaction across the thickness δ. If h_I is appreciably far from the bottom of the container, then we can assume that in phase A the photoreaction has reached local equilibrium, such that no photoreaction occurs, resulting in quasi-steady state mass transport for the interface. The location of the interface therefore advances with a quasi-steady velocity $\boldsymbol{v}_I = dh_I/dt$, where h_I is the height of the interface.

The general boundary condition for mass transfer of chemical species *i* across an interface between a general fluid phase A and fluid phase B in vectorial form is given by a molar mass balance of species *i* at the interface, and can be written without loss of generality:[4]

$$\frac{dN_{I,i}}{dt} = \left\{ \left[\boldsymbol{j}_{A,i} + N_{A,i}(\boldsymbol{v}_A - \boldsymbol{v}_I) \right]_I - \left[\boldsymbol{j}_{B,i} + N_{B,i}(\boldsymbol{v}_B - \boldsymbol{v}_I) \right]_I \right\} \cdot \widehat{\boldsymbol{n}}_I - r_{i,I} \tag{S1}$$

where $N_{X,i}$ is the concentration of species *i* in phase X, $\boldsymbol{j}_{X,i}$ is the diffusive flux of species *i* in phase X, and $\boldsymbol{v}_X$ is the fluid velocity in phase X. $\widehat{\boldsymbol{n}}_I$ is the outward pointing normal vector (defined here as pointing into phase B), and subscript *I* denotes that the quantity is evaluated at the interface. Here, $\boldsymbol{v}_I$ is the velocity of the location of the interface, which in general need not be equal to $\boldsymbol{v}_{A,I}$ or $\boldsymbol{v}_{B,I}$ because it represents the rate of change in location of the interface rather than a fluid velocity. The curly bracketed term represents the total net molar flux of species *i* to/from the interface (either from diffusion or convection), and $r_{i,I}$ is the net reaction rate (in units of $mol\ (volume \cdot time)^{-1}$) of species *i.*

We now make a number of other simplifying assumptions for our bleaching system. Because we already assumed that the interface remains quasi-steady, the left hand side of Eq. (S1) is identically zero. Momentum continuity across the fictitious interface requires that $\boldsymbol{v}_{A_I} = \boldsymbol{v}_{B_I} = \boldsymbol{v}_{\boldsymbol{fluid}}$. Furthermore, we assume DASA is dilute in solution, such that Fick's law can be used for $\boldsymbol{j}_{X,i}$. Finally, we will assume that the bleaching front is symmetric about the vertical centerline of the container, and restrict our analysis to the centerline, such that the problem reduces to a one-dimensional problem in the vertical (negative-*z*) direction.

Under these assumptions and applying the interfacial balance to open DASA 3.0, Eq. (S1) simplifies to:

$$0 = \left(N_{A,open} - N_{B,open}\right)\left(\boldsymbol{v}_{fluid} - \boldsymbol{v}_I\right) + \left(\frac{\partial D_{open} N_{A,open}}{\partial z}\bigg|_I - \frac{\partial D_{open} N_{B,open}}{\partial z}\bigg|_I\right) + \delta r_{open,I} \quad \text{(S2)}$$

The first term in Eq. (S2) represents the net convection of DASA to the interface due to the convective flow. The second term represents the net diffusion of DASA to the interface due to concentration gradients in the bleached and unbleached regions. We expect that such gradients will be small, such that the diffusive flux is negligible compared to the convective flux and will be ignored in subsequent analysis. Making this assumption and solving for the interface velocity gives

$$\boldsymbol{v}_I = \boldsymbol{v}_{fluid} + \frac{\delta r_{I,open}}{\Delta N_{I,open}} \quad \text{(S3)}$$

where $\Delta N_{I,open} = N_{A,open} - N_{B,open}$ is the change in open DASA concentration across the interface. Note that Eq. (S3) assumes nothing about the rate of reaction (or, for that matter, the illumination), and so it will apply to any general reacting system that creates a quasi-steady reaction front of the sort that we have assumed here. To apply this prediction specifically to the DASA system, we use the expression for the net rate of photochemical reaction developed by Lui *et al.*,[5]

$$r_{I,open} = -\sigma\left(N_{I,open}\right)\phi_{OC}\left(N_{I,open}\right)I(h_I)N_{I,open} + k_{back}\left(N_{I,open}\right)\left[N_o - N_{I,open}\right] \quad \text{(S4)}$$

where σ is the absorption cross-section, ϕ_{OC} the quantum efficiency for photoconversion between the open and closed form, $I(h_I)$ the irradiation intensity at the bleaching front, k_{back} the rate constant for the backward photoconversion, and N_o the total concentration of DASA (including open and closed forms) in solution, which remains constant everywhere because the photoreaction is unimolecular. Eq. (S4) explicitly notes the functional dependence on N_{open} of the various photokinetic parameters (these will be suppressed in the equations to follow). Note that, because our analysis treats the bleaching front as a concentration discontinuity, the concentration of the interface $N_{I,open}$ at which the rate is evaluated remains undefined. However, since we have already assumed that no net photoreaction occurs in the bleaching zone, a reasonable choice would be $N_{I,open} = N_{A,open} - c\Delta N_{I,open}$, where c is either 0 or 0.5.

Substituting Eq. (S4) into Eq. (S3) gives

$$\boldsymbol{v}_I = \boldsymbol{v}_{fluid} + \frac{\delta}{\Delta N_{I,open}}\left\{\sigma\phi_{OC}I(h_I)N_{I,open} - k_{back}\left[N_o - N_{I,open}\right]\right\} \quad \text{(S5)}$$

From this equation, it may appear at first glance that we have lost any time dependence of the problem. However, many of the parameters (chiefly $\boldsymbol{v}_{fluid}$ and $I(h_I)$) are implicitly time dependent, both because the bleaching front is advancing through a non-uniform velocity and irradiation field, and because these fields themselves are time-varying. Therefore, solving Eq. (S5) to obtain the entire time-dependence of the front velocity requires solving the full complex coupled mass, heat and flow transport problem, which is not presented in its entirety here.

Supplementary Note 3

Nonzero fluid velocities seen at time t = 0 s in Fig. 5a are likely the result of our imaging set up. Though solutions containing DASA are allowed to settle for two minutes prior to irradiation, as soon as the cuvette is exposed to the white light, it takes several hundreds of milliseconds before the digital camera

used in this experiment is able to adjust to the new brightness in its field of view. As such, the particle image velocimetry (PIV) analyses begin only as the camera has adjusted to the light, at which time the photoreaction is already under way—resulting in a nonzero fluid velocity.

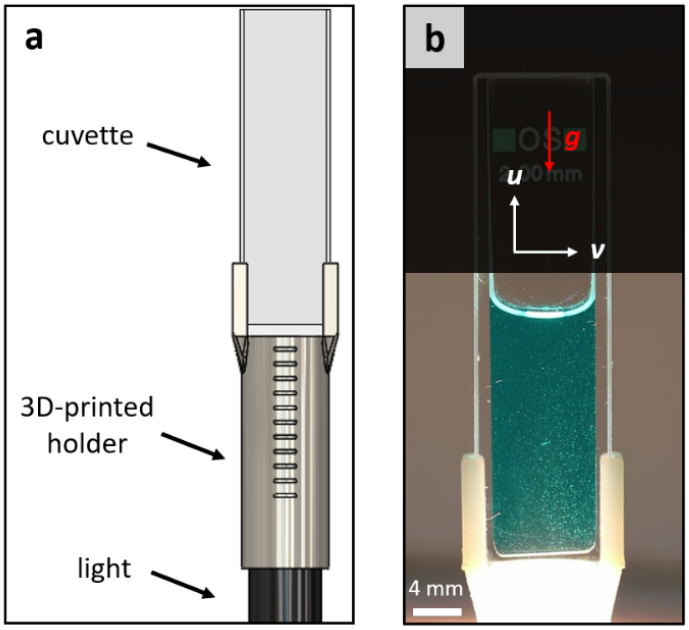

Supplementary Figure 1 | Experimental setup used for UV-vis cuvette studies. a, Labeled SolidWorks rendering of setup. **b**, Image of setup with *u*- and *v*-coordinates labeled. As well as the gravity vector, *g*.

a

b

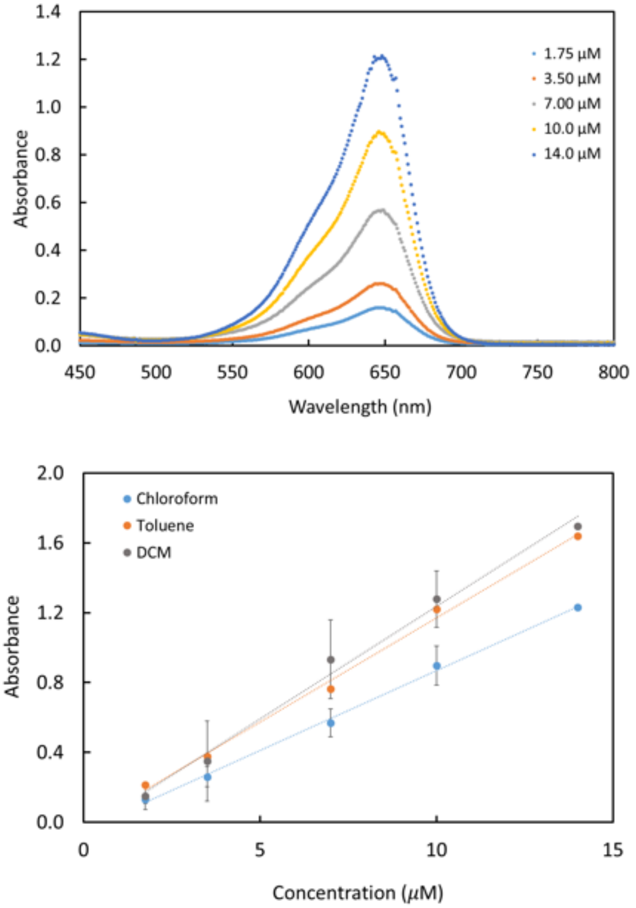

c

Solvent	ε ($M^{-1}\ cm^{-1}$)
Toluene	118,820 $\pm$ 516
Chloroform	88,670 $\pm$ 300
DCM	129,190 $\pm$490

Supplementary Figure 2 | Molar Absorptivity of DASA in toluene, chloroform and dichloromethane. a, Representative absorption spectra obtained with UV-Vis spectrophotometry for various concentrations of DASA in chloroform. Maximum absorption is plotted for each concentration in toluene, chloroform and dichloromethane **b,** from which the slope of the line is used to determine molar absorptivity according to Beer's law ($A = \varepsilon bc$, where ε is molar absorptivity, b is path length and c is concentration). **c,** Molar absorptivity values are reported for all solvents in the table.

a

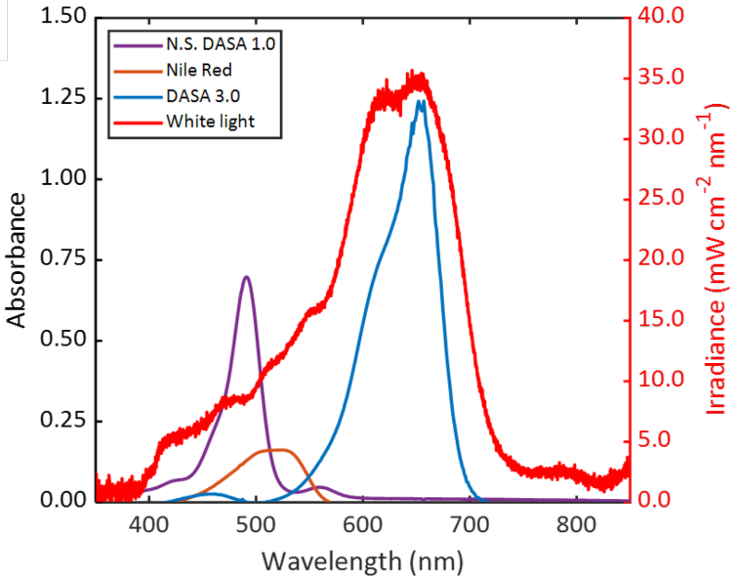

b

Dye (in Toluene)	ε (M^{-1} cm^{-1})
DASA 3.0	118,800 $\pm$ 520
Non-bleaching DASA	70,500 $\pm$ 350
Nile Red	38,000 $\pm$ 500

Supplementary Figure 3 | Molar Absorptivity comparison of high-absorbing dyes and DASAs.
a, (Left axis) Representative absorption spectra obtained with UV-Vis spectrophotometry for 10 µM of DASA 3.0, non-switching DASA 1.0 (see Figure S8 for structure) and Nile red in toluene. (Right axis) Absolute irradiance measurement of the white light source using a USB 4000-UV-Vis spectrometer (OceanOptics, Inc., USA). **b,** Molar absorptivities are determined at maximum absorption for each dye and reported for comparison.

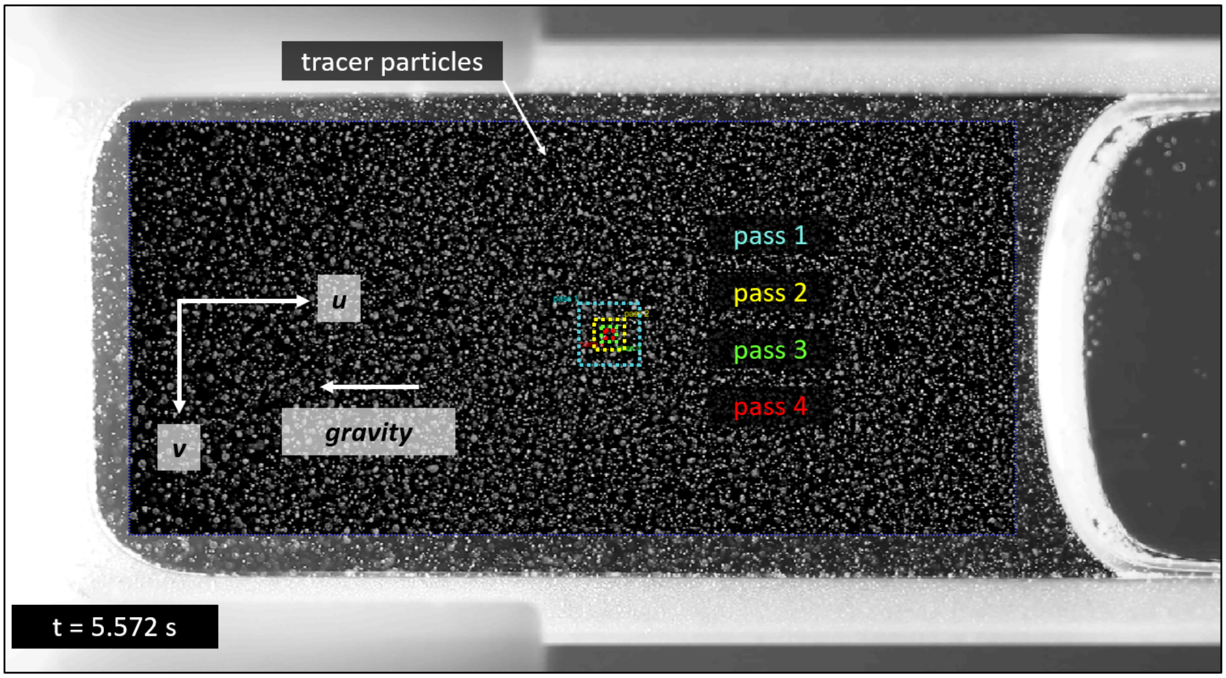

Supplementary Figure 4 | Region of interest and window size selection, as executed in PIVLab. Speckles are tracer particles in space.

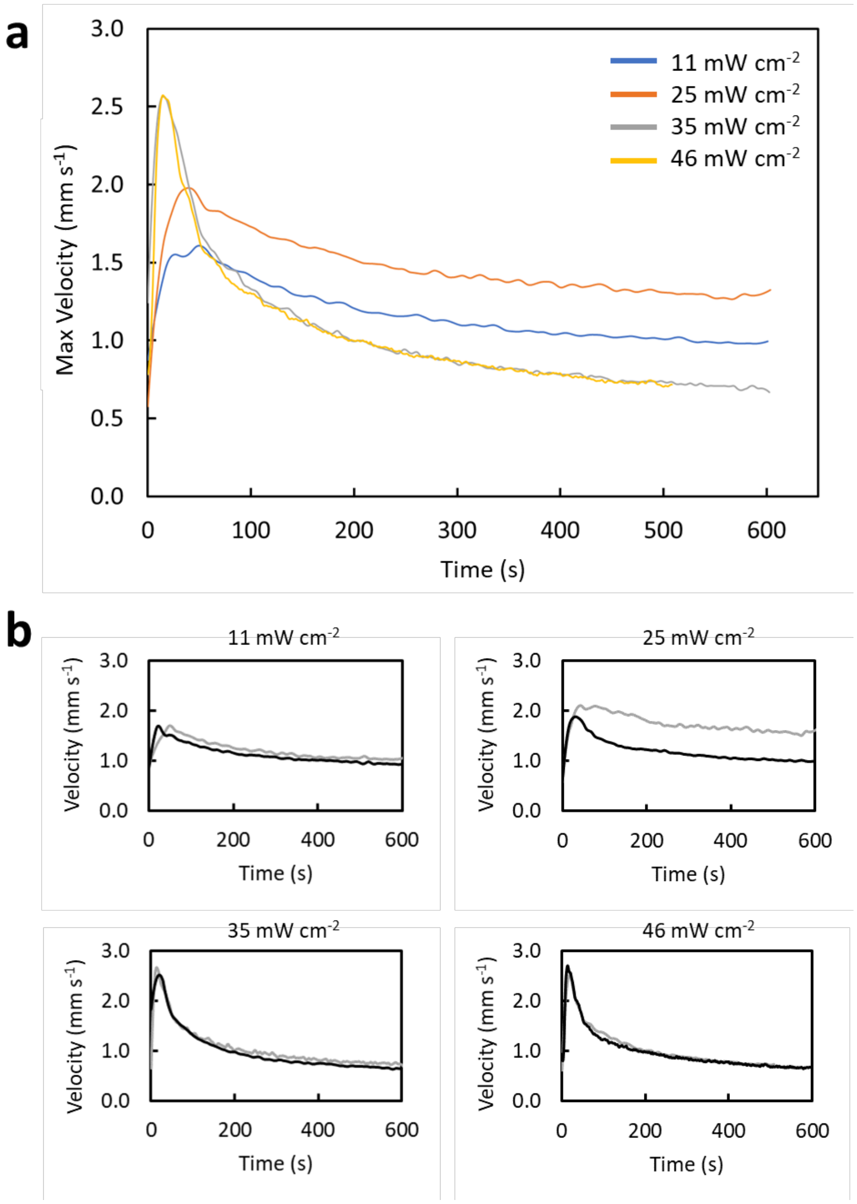

Supplementary Figure 5 | Maximum velocity data for 0.25 mM DASA 3.0 in chloroform irradiated at varying intensities. a, Average curves of differing intensity. **b**, Duplicate runs at each light intensity.

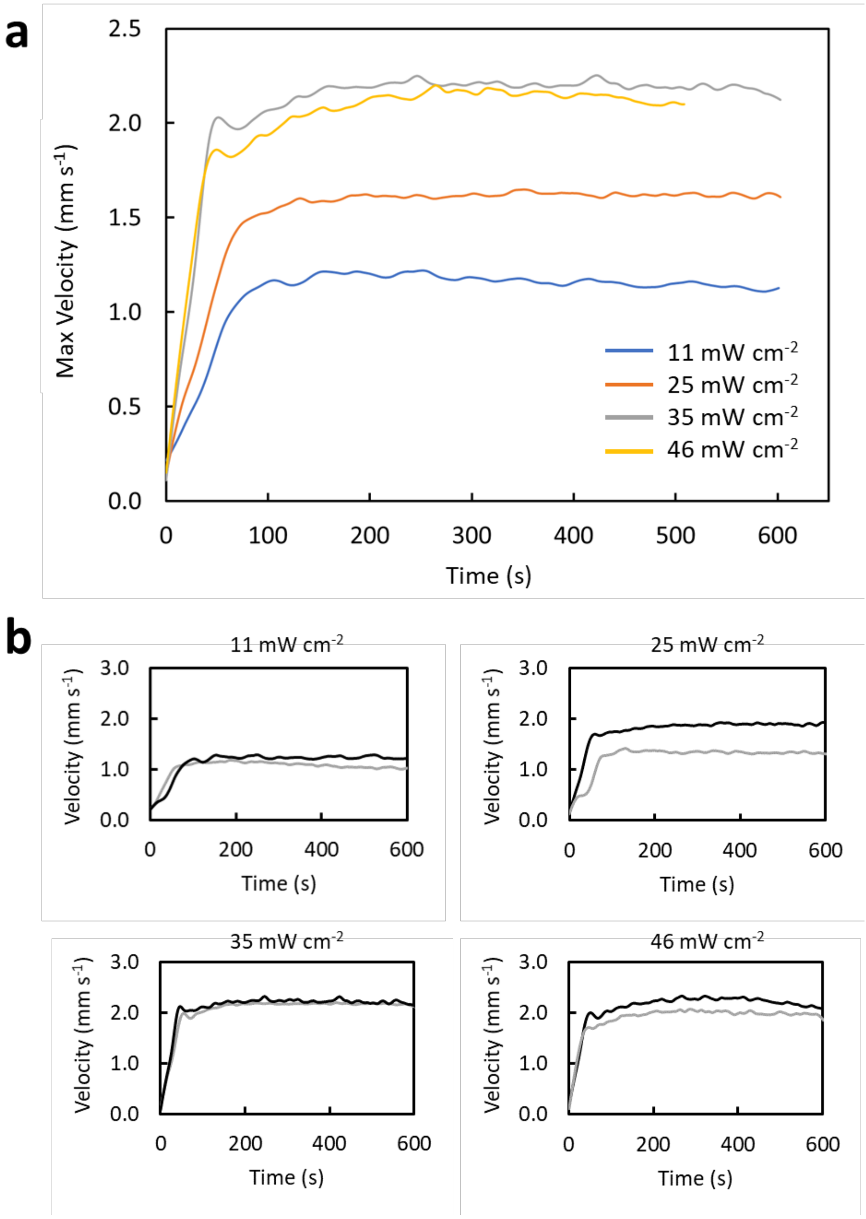

Supplementary Figure 6 | Maximum velocity data for 0.25 mM DASA in toluene irradiated at varying intensities. a, Average curves of differing intensity. **b**, Duplicate runs at each light intensity.

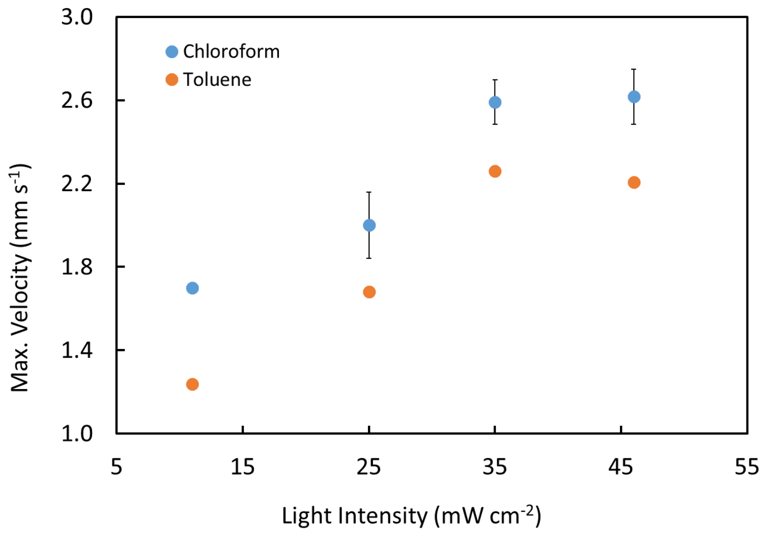

Supplementary Figure 7 | Maximum velocity achieved vs. light intensity for DASA in chloroform and toluene. Error bars: standard deviation, N=2. Note that in some cases the error bars are smaller than the marker size.

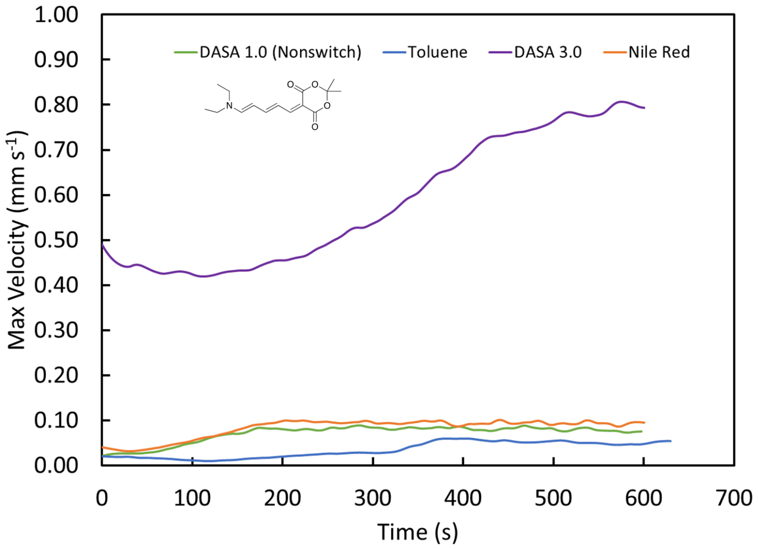

Supplementary Figure 8 | DASA 3.0 and dye comparison. Velocity profiles for 0.25 mM DASA 3.0, Nile red and Non-switching DASA in toluene at 6.34 mW cm^{-2}. For the DASA 3.0-containing solutions, we measured a non-zero flow speed at the earliest times. This rapid increase, which sometimes appears to be instantaneous (as here), is observed in many of the measurements of fluids containing photochromic dyes (e.g., see Figures 5C, S5, and S6), and we suspect is due to the extremely rapid thermal response of the solution which quickly drives fluid motion. At this light intensity, it takes some time for the full convective rolling to establish, so we observe an increase in velocity as the full flow field develops. Future studies will enable measurement of the development and dynamics of the full flow field; these details are not fully captured in the maximum velocity reported here. At all times, the magnitude of flow velocity measured using DASA 3.0 is significantly larger than those observed for non-switching dyes or solvent only, as expected.

a

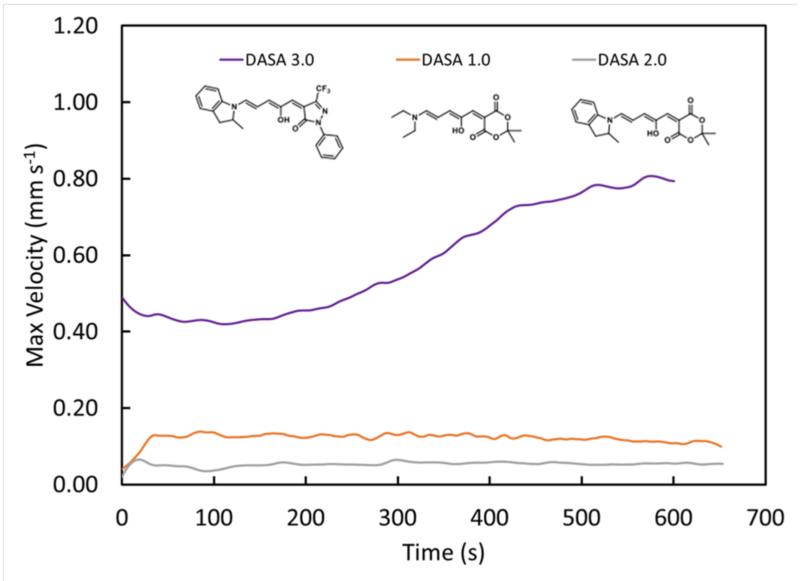

b

DASA	ε (M^{-1} cm^{-1})	% open	Half life $t_{1/2}$	Abs. max
DASA 3.0	118,200	>95%	40s	655 nm
DASA 2.0	107,000	31%	3240s	580 nm
DASA 1.0	102,000	>95%	100s	545 nm

Supplementary Figure 9 | DASA 1.0, 2.0, and 3.0 comparison. a, Velocity profiles of 0.25mM of each generation DASA in toluene at 6.34 mW cm^{-2} light intensity. **b**, Representative details regarding molar absorptivity, thermal equilibria, and half-life ($1/k_b$) of each DASA in toluene.[6] Although a more detailed investigation is required, we attribute the smaller flow velocities developed by DASA 1.0 as compared to DASA 3.0 to the smaller molar absorptivity, as well as the lower wavelength absorption maximum, which is off-peak for the white light source used here (see Figure S3). In the case of DASA 2.0, the equilibrium value and rate of photo conversion from the colored form to colorless form is significantly different: only 31% open for DASA 2.0 as compared to >95% for DASA 1.0 and 3.0, which undermines its ability to serve as a photothermal agent to drive high speed flows.

Supplementary References

1. Thielicke, W. and Stamhuis, E.J. (2014): PIVlab – Towards User-friendly, Affordable and Accurate Digital Particle Image Velocimetry in MATLAB. *Journal of Open Research Software* 2(1):e30, DOI: http://dx.doi.org/10.5334/jors.bl.

2. Thielicke, W. and Stamhuis, E. J. (2014): PIVlab - Time-Resolved Digital Particle Image Velocimetry Tool for MATLAB (version: 2.01). http://dx.doi.org/10.6084/m9.figshare.1092508.

3. Thielicke, William. (2019, April 28). PIVLab Tutorial. Retrieved from https://pivlab.blogspot.com/p/blog-page_19.html.

4. Lui, B. F. et al. Unusual concentration dependence of the photoisomerization reaction in donor–acceptor Stenhouse adducts. *Photochem. Photobiol. Sci.* 1587–1595 (2019).

5. Leal, L G. Advanced Transport Phenomena: Fluid Mechanics and Convective Transport Processes. Cambridge: Cambridge University Press. (2007).

6. Hemmer, J. R. et al. Controlling Dark Equilibria and Enhancing Donor-Acceptor Stenhouse Adduct Photoswitching Properties through Carbon Acid Design. J. Am. Chem. Soc. **140**, 10425–10429 (2018).
