# Supplementary material for: Self-regulating photochemical Rayleigh-Bénard convection using a highly-absorbing organic photoswitch
Source: Nat Commun. 2020 May 25;11:2599. doi: 10.1038/s41467-020-16277-7 (PMC7248117; doi:10.1038/s41467-020-16277-7)
Supplement: Supplementary file 2 — Description of Additional Supplementary Files [file 41467_2020_16277_MOESM2_ESM.pdf]

Description of Additional Supplementary Files

File Name: Supplementary Movie 1
Description: **Bleaching front of 0.25 mM DASA in chloroform.** Cuvette was irradiated using 41.7 mW cm-2 of white light shone through a bandpass filter (λ = 650 ± 40 nm).

File Name: Supplementary Movie 2
Description: **Convection of 0.25 mM DASA in toluene (video speed, 10x).** Left panel depicts the video recording of the convection. Right panel depicts a heat map video of the same video, generated via particle image velocimetry analysis (Canon EOS Rebel T5i, 100mm f/2.8 macro lens, magnification 1x).

File Name: Supplementary Movie 3
Description: **Localized fluid motion in 0.25 mM DASA in chloroform.**
